# Prevalence, resistance profile, and molecular epidemiology of extended-spectrum β-lactamases producing *Escherichia coli* from captive giant pandas

**DOI:** 10.1016/j.onehlt.2026.101513

**Published:** 2026-07-12

**Authors:** Xia Yan, Lucai Wang, Huanrong Zhang, Mei Yang, Bingyu Xue, Ruoshui Zhao, Lin Li, Junjin Xie, Songrui Liu, Xueyang Fan, Xiaoyan Su

**Affiliations:** a Chengdu Research Base of Giant Panda Breeding, The Conservation of Endangered Wildlife Key Laboratory of Sichuan Provincial, Chengdu, China; b College of Veterinary Medicine, Henan Agricultural University, Zhengzhou, China; c College of Animal and Veterinary Sciences, Southwest Minzu University, Chengdu, China; d Brookline High School, Brookline, USA

**Keywords:** Giant panda, ESBL-producing *E. coli*, Antibiotic resistance, Antibiotic resistance genes, Molecular epidemiology

## Abstract

**Background/objective:**

Extended-spectrum β-lactamase (ESBL)-producing *Escherichia coli* (*E. coli*), a bacterium resistant to most β-lactam antibiotics, is a critical clinical global health concern, posing significant health risks to humans and animals including giant pandas. The Chengdu Research Base of Giant Panda Breeding (CRBGP) has the world's largest captive population of giant pandas. This study aimed to investigate the prevalence, antibiotic resistance characteristics, and molecular epidemiology of ESBL-producing *E. coli* among captive giant pandas at the CRBGP.

**Methods:**

ESBL production was screened in 100 *E. coli* isolates from 100 individual giant pandas (different ages and sexes) using the Clinical and Laboratory Standards Institute (CLSI) double-disc combination test. ESBL isolates were subjected to antimicrobial susceptibility testing of 34 antibiotics using the Kirby-Bauer disk diffusion susceptibility test (K-B). Whole genome sequencing (WGS) was performed to characterize genotypes, antibiotic resistance genes (ARGs), mobile genetic elements (MGEs), and multilocus sequence typing (MLST), and the molecular epidemiology of the isolates was further investigated using MLST and the goeBURST algorithm.

**Results:**

Twenty-nine ESBL-producing *E. coli* strains were identified (29.0%, 29/100), representing a marked increase from the 8% prevalence reported during 2020–2021. All 29 isolates exhibited high resistance to β-lactam antibiotics, with 100.0% resistance to amoxicillin, ampicillin, cefazolin, cefuroxime and cefotaxime. A total of 120 different ARG subtypes and 19 ESBL gene subtypes were detected; *bla*_CTX-M-4_ was the most prevalent (100.0%), followed by *bla*_SHV-1_ (96.6%), *bla*_CTX-M-1_ and *bla*_CTX-M-3_ (93.1% each). Analysis of MGEs revealed high carriage rates of *IS26* (89.7%), *intI1* (89.7%), and the conjugation-associated gene *traA* (51.7%). MLST identified 10 sequence types (STs) and one clonal complex (CC1), with ST132 as the founder. ST595 and ST973 were the most common STs (each *n* = 7).

**Conclusions:**

The prevalence of ESBL-producing *E. coli* in captive giant pandas at the CRBGP has risen sharply (29.0%), with high-level multidrug resistance (MDR), a large ARG repertoire, and abundant MGEs indicative of strong horizontal gene transfer (HGT) potential. The presence of shared STs with other hosts suggests potential interspecies transmission. These findings underscore the urgent need for enhanced antimicrobial stewardship and continuous One Health surveillance to protect giant pandas and the broader ecosystem.

## 1 Introduction

*Escherichia coli* (*E. coli*) is a Gram-negative commensal bacterium and also an important opportunistic pathogen that may pose serious health risks for humans and animals. ESBL-producing *E. coli*, a bacterium resistant to most β-lactam antibiotics, is listed among the highest-priority pathogens by the World Health Organization (WHO) in the context of One Health [1], [2]. The emergence of ESBL-producing *E. coli* has increased worldwide in recent years, which seriously compromises the effectiveness of antimicrobial therapy and increases treatment difficulty [3]. Previous studies have demonstrated that mortality associated with ESBL-producing *E. coli* bacteremia is significantly higher than that associated with non-ESBL-producing strains [4]. Antimicrobial misuse is a significant risk factor for the emergence of ESBL-producing *E. coli.* Furthermore, HGT via MGEs such as integrons, transposons, integrative conjugative elements, genomic islands, and plasmids plays a key role in the spread of ARGs carried by ESBL-producing *E. coli* [5], [6], [7]. In addition, MLST has become a powerful tool for genotyping specific bacterial species. MLST utilizes internal fragments of multiple housekeeping genes and the combination of each allele defines the sequence type for each isolate, which is usually used to investigate the phylogeny and population structure of different bacterial isolates, and facilitates the understanding of bacterial molecular epidemiology [8], [9].

MLST has also been applied to investigate the molecular epidemiology of *E. coli* isolates from endangered wildlife, including the giant panda. The giant panda (*Ailuropoda melanoleuca*) is one of the world's most recognized vulnerable species and is only distributed in the mountainous areas of Sichuan, Gansu, and Shanxi provinces in China [10]. Supported by in-situ and ex-situ conservation, giant panda populations have gradually increased. However, giant pandas are still threatened by bacterial diseases, especially the diseases caused by intestinal pathogens [11]. *E. coli* is the bacterial species most frequently detected in the intestinal tract of giant pandas [12]. Certain strains of *E. coli* and other intestinal bacteria can become opportunistic pathogens, leading to gastrointestinal infections under specific conditions. The pathogenic effect of *E. coli* on giant pandas is mainly manifested as diarrhea symptoms in clinical practice, which can lead to death in severe cases. Chen et al. [13] first reported the death of giant pandas infected with Enteropathogenic *E. coli* in 1983; Xiong et al. isolated and identified an *E. coli* (EL-1) strain from dead sub-adult giant pandas [14]. Antibiotic therapy is still used clinically for bacterial infection of giant pandas. Over the past few years, numerous studies have shown that ESBL-producing *E. coli* strains have emerged in the giant panda population, showing multi-drug resistance and carrying multiple ARGs [15], [16], [17], [18]. By 2024, the number of captive giant pandas in the CRBGP had reached 244. It maintains the largest captive population worldwide. The base is equipped with specialized facilities including delivery houses, feeding areas, research laboratories, and a panda hospital. Environmental enrichment measures, such as simulating natural habitats, providing diverse feeding and resting areas, and regular health monitoring, are routinely implemented to ensure the well-being of the animals. It is necessary to understand the prevalence and resistance characteristics of ESBL-producing *E. coli* in this population, which is essential for the development of effective disease prevention and antimicrobial management strategies in the clinical diagnosis and treatment of giant pandas at CRBGP.

In this study, 100 *E. coli* strains were isolated from the fresh feces of 100 captive giant pandas of different ages and sexes. Subsequently, ESBL production was assessed in all 100 *E. coli* isolates, and the antimicrobial resistance phenotypes, ARGs, and MLST typing of ESBL-positive strains were further analyzed. The aim of this study was to understand the resistance characteristics and molecular epidemiology of ESBL-producing *E. coli* strains in the giant panda population within CRBGP, providing evidence-based support for antimicrobial stewardship and clinical decision-making in giant pandas.

## 2 Materials and methods

### 2.1 Bacterial source and screening for ESBL phenotype

One hundred *E. coli* isolates (one isolate per fresh fecal sample) were collected from 100 captive giant pandas housed at the CRBGP (Sichuan Province, China) in 2023. At the time of sampling, the total captive population was approximately 237. However, 30–40 giant pandas were temporarily absent from the CRBGP, some animals had received antimicrobial treatment within the preceding three months, and some fecal samples did not isolate *E. coli* strains through culture. Therefore, only giant pandas that met the following inclusion criteria were enrolled: (i) clinically healthy at the time of sampling, (ii) no documented antimicrobial exposure within the previous three months and (iii) successful isolation of *E. coli* from fresh feces. A total of 100 *E. coli* isolates from 100 different pandas were obtained accordingly. The sampled pandas comprised 60 females and 40 males, categorized into four age groups: geriatric (≥20 years, *n* = 7), adult (5–19 years, *n* = 55), sub-adult (1.5–5 years, *n* = 27), and juvenile (0–1.5 years, *n* = 11). All animals were managed under routine husbandry practices at CRBGP. These isolates were identified as *E. coli* by Gram staining, biochemical testing, and 16S rRNA gene sequencing. All isolates were screened for ESBL production using the CLSI-recommended double-disk synergy test (DDST) with ceftazidime (CAZ) and cefotaxime (CTX) alone and in combination with CAL. ESBL production was phenotypically confirmed by detecting an increase in the inhibition zone diameter of the β-lactam disk when combined with a clavulanate disk compared with the β-lactam disk alone. If the enhancement value was >5 mm, the isolate was classified as ESBL-producing.

### 2.2 Antimicrobial susceptibility testing of ESBL-producing *E. coli*

Antimicrobial susceptibility testing of ESBL-producing *E. coli* strains for 34 antibiotics was determined in triplicate using the K-B recommended by the CLSI. Seven classes of antimicrobial agents from Hangzhou Microbial Reagent Co., Ltd. (China) were tested, as follows: β-lactams: amoxicillin (AMX, 20 μg), piperacillin (PIP, 100 μg), meropenem (MEM, 10 μg), imipenem (IPM, 10 μg), aztreonam (AT, 30 μg), sultamicillin (AMS, 10 μg), tazocin (TZP, 100 μg), cefamezin (CZ, 30 μg), cephalexin (CA, 30 μg), cefuroxime (CXM, 30 μg), cefoxitin (FOX, 30 μg), ceftriaxone (CTR, 30 μg), ceftazidime (CAZ, 30 μg), cefepime (FEP, 30 μg), cefotaxime (CTX, 30 μg), ampicillin (AM, 10 μg), cefotaxime/clavulanic acid (CTC, 30/10 μg). Aminoglycosides: kanamycin (K, 30 μg), gentamicin (GM, 10 μg), streptomycin (S, 10 μg), neomycin (N, 30 μg), amikacin (AK, 30 μg). Quinolones: norfloxacin (NOR, 10 μg), levofloxacin (LVX, 5 μg), ciprofloxacin (CIP, 5 μg), enrofloxacin (ENR, 10 μg). Chloramphenicol: chloramphenicol (C, 30 μg), florfenicol (FON, 30 μg), macrolides: azithromycin (AZM, 15 μg). Tetracyclines: doxycycline (DX, 30 μg), minocycline (MI, 30 μg), tetracycline (TE, 30 μg). Sulfonamides: compound sulfamethoxazole (SXT, 23.75/1.25 μg), trimethoprim (TMP, 5 μg). Antibiotics were selected based on clinical records provided by CRBGP veterinarians and previous studies on the resistance of giant pandas (Yan et al., 2021; Guo et al., 2015; Zou et al., 2018). *E. coli* ATCC 25922 was used as a control for antimicrobial susceptibility testing.

### 2.3 DNA extraction for the ESBL-producing *E. coli* isolates

Total genomic DNA was extracted from the ESBL-producing *E. coli* isolates based on the above antimicrobial susceptibility test results using the TIANamp Bacteria DNA kit (Tiangen Biotech, Beijing, China) according to the manufacturer's instructions. The quality of DNA was checked by spectrophotometric analysis using NanoDrop ND-2000 (Nanodrop, Wilmington, DE, USA). The DNA samples were stored at −20 °C until use.

### 2.4 Whole-genome sequencing and identification of ESBL genotypes, ARGs, and MGEs carried by the ESBLs *E. coli* isolates from giant pandas

To evaluate the ESBL genotypes, ARGs and MGEs carried by the ESBL-producing *E. coli* isolates in this study, WGS was performed by Lianchuan Biotechnology Co., Ltd. (Hangzhou, China) on an Illumina NovaSeq 6000 platform, generating 2 × 150 bp paired-end reads. Raw sequencing reads were subjected to stringent quality control using fastp (version 0.23.2) with the following criteria: removal of read pairs if either read contained >10% undetermined bases (N) or > 50% of bases had a Phred quality score ≤ 5. After filtering, high-quality clean data were obtained, with Q30 values exceeding 96.0%. The subsequent de novo genome assembly was performed using Unicycler (version 0.4.8) with default parameters. ARGs were identified using ResFinder 4.1 combined with the Comprehensive Antibiotic Resistance Database (CARD). β-lactamase gene variants were further analyzed using the β-lactamase database (BLDB). MGEs, including insertion sequences (IS), transposons, and integrons were detected using ISfinder, TnNumber, and IntegronFinder, respectively, with default parameters.

### 2.5 The molecular epidemiology analysis of the ESBL-producing *E. coli* isolates using MLST

MLST was performed by extracting the allele sequences of eight standard housekeeping genes (*dinB*, *icdA*, *pabB*, *polB*, *putP*, *trpA*, *trpB*, and *uidA*) from the WGS data, according to the Pasteur Institute MLST scheme (https://bigsdb.pasteur.fr/ecoli/). A minimum spanning tree was constructed with PHYLOViZ 2.0 software based on the goeBURST distance algorithm, using the allelic profiles obtained in this study. Isolates sharing identical alleles at seven of the eight loci were defined as a clonal group. A clonal complex (CC) was defined as a group containing four or more sequence types (STs), within which one ST was identified as the founder ST; the other STs were considered its evolutionary derivatives. A single-locus variant (SLV) differs from the founding ST at only one housekeeping locus. STs that did not belong to any clonal complex were designated as singletons.

### 2.6 Statistical analysis

Data were analyzed by GraphPad Prism v8 (GraphPad Software, USA). Differences in prevalence rates were evaluated using the chi-square test or Fisher's exact test. Antimicrobial susceptibility and ARG carriage among ESBL-producing *E. coli* isolates were compared across different age groups and sexes. Statistical significance was defined as *P* < 0.05.

## 3 Results

### 3.1 Prevalence of ESBL-producing *E. coli* isolates

**Fig. 1:**
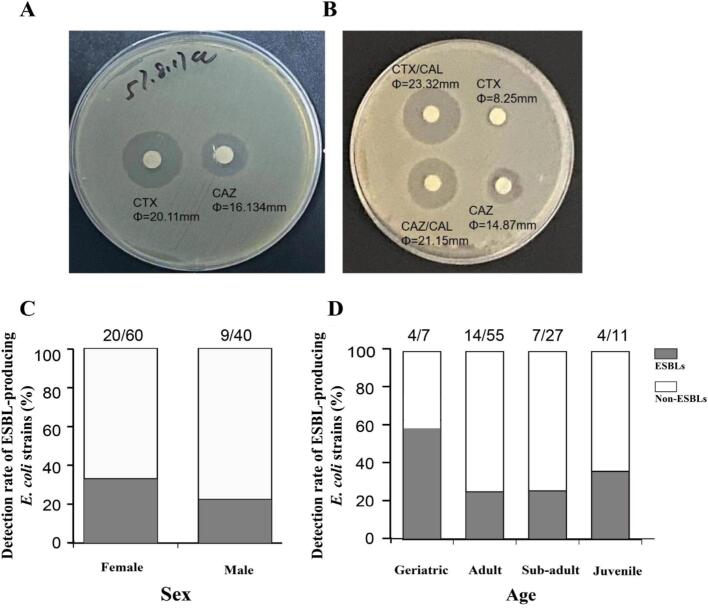
The detection of ESBL-producing *E. coli* strains isolated from giant pandas of different sexes and ages. A. The preliminary screening test of ESBL-producing *E. coli*. When the inhibition zone diameter of CTX is ≤27 mm and CAZ is ≤22 mm, it is suggested that this *E. coli* isolate may produce ESBLs. B. The confirmation test for ESBL-producing *E. coli*. When the inhibition zone diameters difference between CTX and CTX/CAL or CAZ and CAZ/CAL is ≥5 mm, it is suggested that this *E. coli* isolate produces ESBLs. CRO (ceftriaxone), CTX (cefotaxime), CAZ (ceftazidime), CTX/CAL (cefotaxime/clavulanate), CAZ/CAL (ceftazidime/clavulanate). C. The detection rate of ESBL-producing *E. coli* strains isolated from giant pandas of different sexes. D. The detection rate of ESBL-producing *E. coli* strains isolated from giant pandas of different ages. Geriatric: giant pandas aged 20 years or older; Adult: giant pandas aged 5–19 years; Sub-adult: giant pandas aged 1.5–5 years; Juvenile: giant pandas aged 0–1.5 years.

Twenty-nine ESBL-producing *E. coli* strains were detected through the double-disk synergy test with a prevalence of 29.0% (*n* = 29/100) in the 100 strains from 100 giant pandas, with detection rates of 33.3% in females (*n* = 20/60), compared with 22.5% in males (*n* = 9/40). Sex was not significantly associated with ESBL prevalence (*P* > *0.05*). The detection rate of ESBL-producing *E. coli* was 57.1% (*n* = 4/7) in the geriatric group, 25.6% (*n* = 14/55) in the adult group, 25.9% (*n* = 7/27) in the sub-adult group, and 36.4% (n = 4/11) in the juvenile group. There was no significant difference in the detection rate among age groups of the giant pandas (*P* > *0.05*) (Fig. 1).

### 3.2 The genotypes of ESBL-producing *E. coli* isolates

**Fig. 2:**
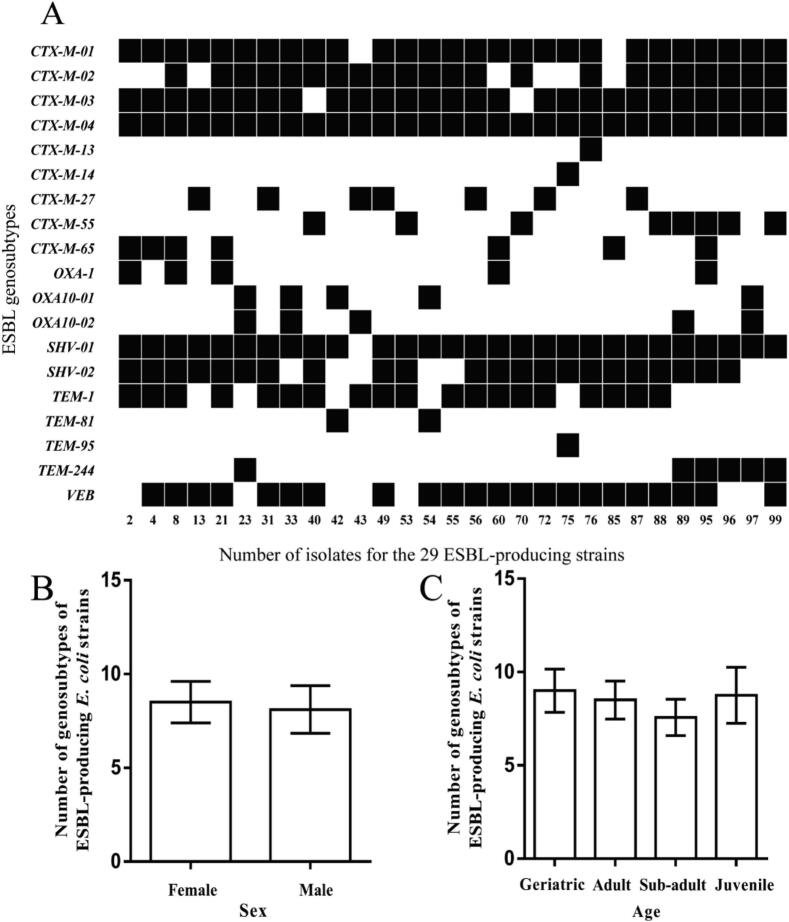
The genosubtypes of ESBLs in the 29 ESBL-producing *E. coli* strains isolated from giant pandas. A. Presence–absence heatmap of ESBL genotypes detected by WGS. Columns: Number of 29 ESBL-producing *E. coli* strains. Rows: 19 different ESBL genotypes. White: The ESBL-producing *E. coli* isolates which did not carry the ESBL genotypes; Black: The ESBL-producing *E. coli* isolates which carried the ESBL genotypes. B. The effect of sex of giant pandas on the number of genosubtypes in ESBL-producing *E. coli*. C. The effect of age of giant pandas on the number of genosubtypes in ESBL-producing *E. coli*.

A total of 19 ESBL gene variants were detected through WGS in 29 ESBL-producing *E. coli,* including *bla*_CTX-M-1_, *bla*_CTX-M-2_, *bla*_CTX-M-3_, *bla*_CTX-M-4_, *bla*_CTX-M-13_, *bla*_CTX-M-14_, *bla*_CTX-M-27_, *bla*_CTX-M-55_, *bla*_CTX-M-65_, *bla*_OXA-1_, *bla*_OXA-10-1_, *bla*_OXA-10-2_, *bla*_SHV-1_, *bla*_SHV-2_, *bla*_TEM-1_, *bla*_TEM-81_, *bla*_TEM-95_, *bla*_TEM-244_ and *bla*_VEB_, of which *bla*_CTX-M-4_ was the predominant genotype with a detection rate of 100.0%, followed by *bla*_SHV-1_, *bla*_CTX-M-1_ and *bla*_CTX-M-3_ with detection rates of 96.6% (28/29), 93.1% (27/29) and 93.1% (27/29), respectively, while the remaining ESBL genosubtypes had detection rates ranging from 3.4% (1/29) to 75.9% (22/29) (Fig. 2A). The effects of sex and age of giant pandas on the number of ESBL genosubtypes in the 29 ESBL-producing *E. coli* isolates were further analyzed. It was found that sex and age of giant pandas had no significant influence on the number of ESBL genosubtypes (*P* > 0.05) (Fig. 2B) (Fig. 2C).

### 3.3 Antimicrobial susceptibility analysis of the ESBL-producing *E. coli*

**Fig. 3:**
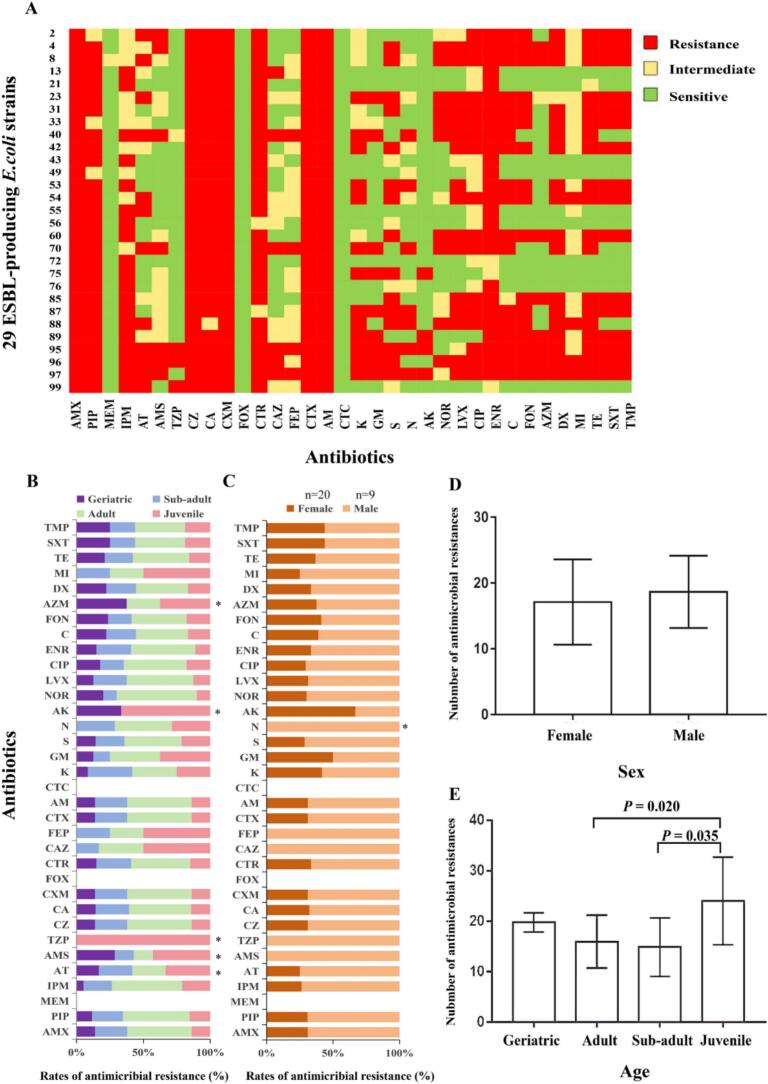
The antibiotics resistance profiles of 29 ESBL-producing *E. coli* isolated from giant pandas. A. Distribution of resistance in 29 ESBL-producing *E. coli* strains. The abscissa represents 34 different antibiotics and the ordinate represents individual isolates. Red: resistant; Yellow: intermediate; Green: sensitive. B. Antibiotic resistance patterns of 29 ESBL-producing *E. coli* isolates from giant pandas of different ages. Significant differences between groups are marked with*. C. Antibiotic resistance patterns of 29 ESBL-producing *E. coli* isolates from giant pandas of different sexes. Significant differences between groups are marked with *. D. Effect of sex of giant pandas on the number of antimicrobial resistances in each giant panda-derived ESBL-producing *E. coli*. E. Effect of age of giant pandas on the number of antimicrobial resistances in each giant panda-derived ESBL-producing *E. coli*. *P* < 0.05: The age and sex of giant pandas had a significant influence on the antibiotic resistance of giant panda-derived ESBL-producing *E. coli*. (For interpretation of the references to colour in this figure legend, the reader is referred to the web version of this article.)

Through antimicrobial susceptibility testing, the 29 ESBL-producing *E. coli* strains had a 100.0% resistance rate to AMX, CZ, CXM, CTX and AM, followed by CA (96.6%), CTR (93.1%), PIP (89.7%), ENR (89.7%); resistance to the remaining 25 antimicrobials was observed in less than 70.0% of the samples (Fig. 3A). Antimicrobial resistance rates of ESBL-producing *E. coli* strains to TZP, CAZ, AK, IPM and AZM were significantly related to age (*P* < 0.05) (Fig. 3B). Antimicrobial resistance rates of ESBL-producing *E. coli* strains to N, FEP, CAZ and AMS were significantly related to sex (*P* < 0.05) (Fig. 3C). We further analyzed the effects of sex and age on the number of antimicrobial resistances in each ESBL-producing *E. coli*. It was found that the number of antimicrobial resistances did not differ significantly between sexes (*P >* 0.05) (Fig. 3D). However, the mean number of antimicrobial resistance phenotypes per isolate in juvenile giant pandas (mean = 24.0, *n* = 4) was significantly higher than that in adult (mean = 15.9, *n* = 14) and sub-adult (mean = 16.0, *n* = 7) giant pandas (*P* < 0.05) (Fig. 3E).

For the 7 antibiotic categories in the study, the resistance rate of 29 ESBL-producing *E. coli* strains to β-lactam antibiotics was the highest (100.0%, 29/29), followed by quinolones (89.7%, 26/29), chloramphenicols (65.5%, 19/29), tetracyclines (65.5%, 19/29), aminoglycosides (62.1%, 18/29), sulfonamides (55.2%, 16/29). The resistance rate to macrolides antibiotics was the lowest (27.6%, 8/29). Phenotypic characterization of antibiotic resistance indicated that 65.5% (19/29) ESBL isolates (E2, E4, E8, E23, E31, E33, E40, E42, E53, E54, E60, E70, E85, E87, E88, E89, E95, E96, and E97) were classified as MDR strains, of which strain E97 was resistant to 29 antibiotics (Table 1).

**Table 1:** Resistance pattern of 29 ESBL-producing *E. coli* isolates from captive giant pandas.

Strains	Age	Sex	Resisrtance profiles	Antibiotic categories	MDR
E2	Geriatric	Female	AMX/AT/AMS/CZ/CA/CXM/CTR/CTX/AM/ENR/C/FON/DX/TE/SXT/TMP	β-Lactams / Quinolones /Chloramphenicols/ Tetracyclines/ Sulfonamides	MDR
E4	Geriatric	Female	AMX/PIP/AMS/CZ/CA/CXM/CTR/CTX/AM/S/NOR/LVX/CIP/ENR/C/FON/AZM/DX/TE/SXT/TMP	β-Lactams /Amionglycosides /Quinolones/ Chloramphenicols/Macrolides/ Tetracyclines/Sulfonamides	MDR
E8	Geriatric	Female	AMX/PIP/AT/CZ/CA/CXM/CTR/CTX/AM/S/NOR/LVX/CIP/ENR/C/FON/AZM/DX/TE/SXT/TMP	β-Lactams /Amionglycosides /Quinolones/ Chloramphenicols/Macrolides/ Tetracyclines/Sulfonamides	MDR
E13	Adult	Female	AMX/PIP/IPM/CZ/CA/CXM/CTR/CAZ/CTX/AM/ENR	β-Lactams/Quinolones	–
E21	Adult	Female	AMX/PIP/IPM/CZ/CA/CXM/CTR/CTX/AM/ENR	β-Lactams/Quinolones	–
E23	Adult	Male	AMX/PIP/AT/CZ/CA/CXM/CTR/CTX/AM/K/GM/S/NOR/LVX/CIP/ENR/C/FON/TE/SXT/TMP	β-Lactams/ Amionglycosides/ Quinolones/Chloramphenicols/ Tetracyclines/Sulfonamides	MDR
E31	Adult	Female	AMX/PIP/CZ/CA/CXM/CTR/CTX/AM/S/NOR/LVX	β-Lactams/ Amionglycosides/ Quinolones/Chloramphenicols/ Tetracyclines/Sulfonamides	MDR
			/CIP/ENR/C/FON/DX/TE/SXT/TMP		
E33	Adult	Male	AMX/CZ/CA/CXM/CTR/CTX/AM/NOR/LVX/	β-Lactams/ Quinolones/ Chloramphenicols/ Tetracyclines/Sulfonamides	MDR
			CIP/ENR/C/FON/DX/TE/SXT/TMP		
E40	Sub-adult	Female	AMX/PIP/IPM/AT/AMS/CZ/CA/CXM/CTR/CAZ/FEP/	β-Lactams/ Amionglycosides /Quinolones/ Chloramphenicols/Tetracyclines	MDR
			CTX/AM/K/GM/N/NOR.LVX/CIP/ENR/C/DX/TE		
E42	Sub-adult	Male	AMX/PIP/CZ/CA/CXM/CTR/CTX/AM/K/S/LVX/	β-Lactams/ Amionglycosides/ Quinolones/Chloramphenicols/ Tetracyclines/Sulfonamides	MDR
			CIP/ENR/C/FON/DX/TE/SXT/TMP		
E43	Sub-adult	Male	AMX/PIP/IPM/CZ/CA/CXM/CTR/CTX/AM/ENR	β-Lactams/Quinolones	–
E49	Sub-adult	Female	AMX/CA/CZ/CXM/CTR/CTX/AM/ENR	β-Lactams/Quinolones	–
E53	Sub-adult	Female	AMX/PIP/IPM/CZ/CA/CXM/CTR/CTX/AM/K/S/N/LVX/	β-Lactams/ Amionglycosides/ Quinolones/Chloramphenicols/ Tetracyclines/Sulfonamides	MDR
			ENR/C/FON/DX/TE/SXT/TMP		
E54	Sub-adult	Male	AMX/PIP/AT/CZ/CA/CXM/CTR/CTX/AM/K/S/LVX/CIP/ENR/C/FON/DX/MI/TE/SXT/TMP	β-Lactams/ Amionglycosides/ Quinolones/ Chloramphenicols/Tetracyclines/Sulfonamides	MDR
E55	Sub-adult	Male	AMX/PIP/IPM/AT/CZ/CA/CXM/CTR/CTX/AM/ENR	β-Lactams/Quinolones	–
E56	Adult	Female	AMX/PIP/IPM/CZ/CA/CXM/CTX/AM/ENR	β-Lactams/Quinolones	–
E60	Adult	Male	AMX/PIP/IPM/CZ/CA/CXM/CTR/AM/S/NOR/LVX/CIP/ENR/C/FON/AZM/DX/TE/SXT/TMP	β-Lactams/ Amionglycosides/ Quinolones/ Chloramphenicols/ Macrolides/ Tetracyclines/Sulfonamides	MDR
E70	Adult	Female	AMX/PIP/AT/AMS/CZ/CA/CXM/CTR/CAZ/FEP/CTX/AM/K/GM/N/NOR/LVX/CTP/ENR/C/DX/TE	β-Lactams/ Amionglycosides/ Quinolones/ Chloramphenicols/Tetracyclines	MDR
E72	Adult	Female	AMX/PIP/IPM/CZ/CA/CXM/CTR/CTX/AM/	β-Lactams	–
E75	Adult	Female	AMX/PIP/IPM/CZ/CA/CXM/CTR/CTX/AM/K/GM/S/AK/	β-Lactams/Amionglycosides	–
E76	Adult	Female	AMX/PIP/IPM/CZ/CA/CXM/CTR/CTX/AM/ENR	β-Lactams/Quinolones	–
E85	Adult	Female	AMX/PIP/IPM/CZ/CA/CXM/CTR/CTX/AM/S/LVX/CIP/CIP/ENR/FON/AZM/DX/TE/SXT/TMP	β-Lactams/ Amionglycosides/ Quinolones/Chloramphenicols/ Macrolides/Tetracyclines/Sulfonamides	MDR
E87	Adult	Female	AMX/PIP/IPM/CZ/CA/CXM/CTX/AM/K/GM/S/N/LVX/CIP/ENR/C/FON/DX/TE/SXT/TMP	β-Lactams/ Amionglycosides/ Quinolones/Chloramphenicols/ Tetracyclines/Sulfonamides	MDR
E88	Adult	Female	AMX/PIP/IPM/AT/CZ/CXM/CTR/CTX/AM/K/S/N/NOR/LVX/CIP/ENR/C/FON/DX/MI/TE	β-Lactams/ Amionglycosides/ Quinolones/Chloramphenicols/Tetracyclines	MDR
E89	Geriatric	Male	AMX/PIP/IPM/CZ/CA/CXM/CTR/CTX/AM/K/GM/AK/	β-Lactams/ Amionglycosides/ Quinolones/ Chloramphenicols/ Macrolides/ Tetracyclines/Sulfonamides	MDR
			ENR/C/FON/AZM/DX/TE/SXT/TMP		
E95	Juvenile	Female	AMX/PIP/IPM/AT/AMS/TZP/CZ/CA/CXM/CTR/CAZ/FEP/CTX/AM/K/GM/S/N/AK/CIP/ENR/C/FON/AZM/DX/TE/SXT/TMP	β-Lactams/ Amionglycosides/ Quinolones/ Chloramphenicols/ Macrolides/ Tetracyclines/Sulfonamides	MDR
E96	Juvenile	Male	AMX/PIP/IPM/AT/AMS/TZP/CZ/CA/CXM/CTR/CAZ/CTX/AM/K/GM/S/NOR/LVX/CIP/ENR/C/FON/AZM/DX/MI/TE/SXT/TMP	β-Lactams/ Amionglycosides/ Quinolones/ Chloramphenicols/ Macrolides/ Tetracyclines/Sulfonamides	MDR
E97	Juvenile	Female	AMX/PIP/IPM/AT/AMS/CZ/CA/CXM/CTR/CAZ/FEP/CTX/AM/K/GM/S/N/AK/LVX/CIP/ENR/C/FON/AZM/DX/MI/TE/SXT/TMP	β-Lactams/ Amionglycosides/ Quinolones/ Chloramphenicols/ Macrolides/ Tetracyclines/Sulfonamides	MDR
E99	Juvenile	Female	AMX/PIP/IPM/AT/TZP/CZ/CA/CXM/CTR/CTX/AM	β-Lactams	–

### 3.4 Prevalence of ARGs and MGEs of the ESBL-producing *E. coli*

**Fig. 4:**
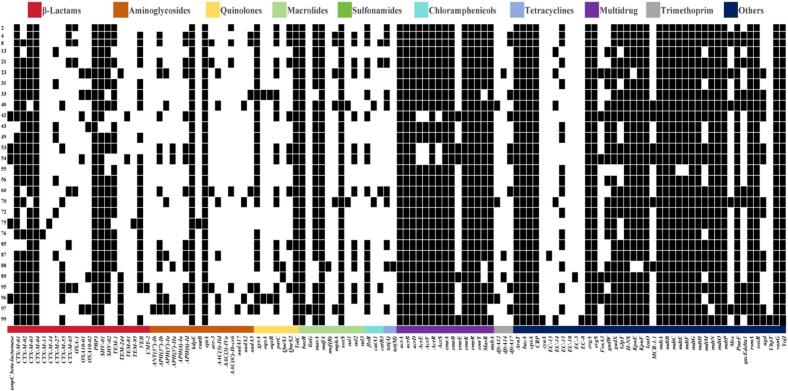
The ARGs profile of 29 ESBL-producing *E. coli* isolated from giant panda. Abscissa is different subtypes of ARGs and the ordinate is isolates individuals.

ARGs were identified in 29 ESBL-producing *E. coli* isolates through WGS. The results showed an abundance of various ARG subtypes. A total of 120 different subtypes were detected, mainly belonging to β-lactam, aminoglycoside, and multi-drug resistance, among which 37 subtypes of ARGs were carried by all 29 ESBL-producing *E. coli* strains*,* such as *acrA*, *bla*_*CTX-M-4*_, *emrA and mdfA* (Fig. 4).

**Fig. 5:**
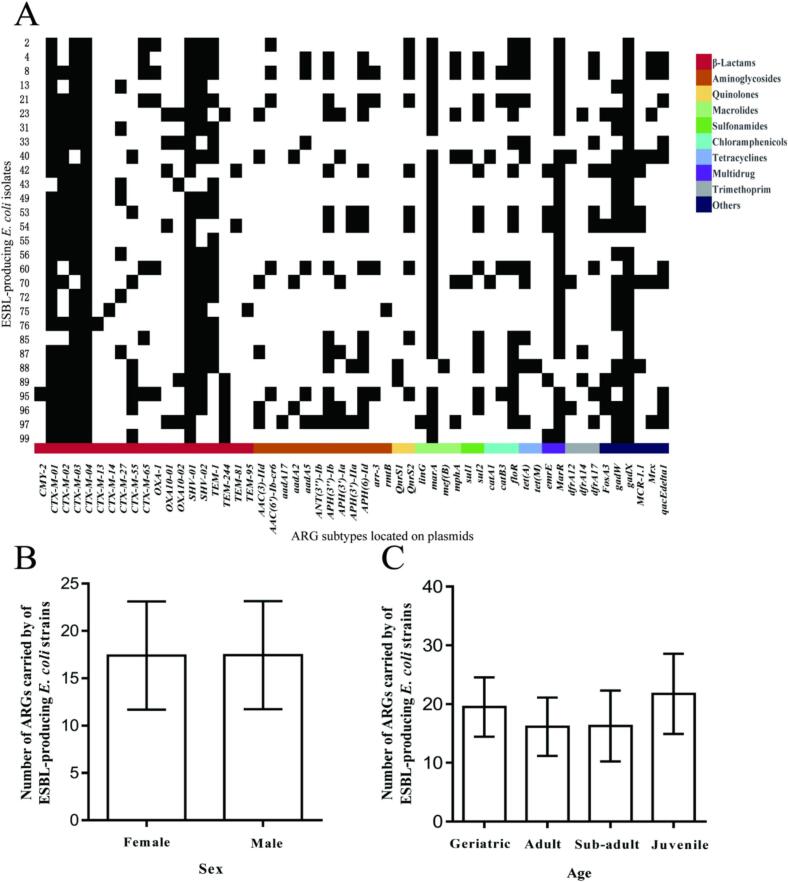
The Profiles of plasmid-borne ARGs of ESBL-producing *E. coli* isolated from giant pandas. A. Distribution of ARGs on the plasmids in 29 ESBL-producing *E. coli* strains isolated from giant pandas. Abscissa is different subtypes of ARGs on the plasmids, and the ordinate is individual isolates. B. Effect of sex of giant panda on the number of ARGs in each giant panda-derived ESBL-producing *E. coli*. C. Effect of age of giant panda on the number of ARGs in each giant panda-derived ESBL-producing *E. coli*.

We further analyzed plasmid-borne ARGs of 29 ESBL-producing *E. coli*, as these genes have the potential for horizontal transmission. It was found that among the 120 subtypes of ARGs, 55 of them were located on plasmids and mainly belonged to β-lactam and aminoglycoside resistance classes. Among them, the carriage rate of *CTX-M-4* was 100%. Notably, except for *VEB*, the remaining 18 ESBL genosubtypes were all located on plasmids (Fig. 5A). These ARG findings were highly concordant with phenotypic resistance (Fig. 3A; Table 1): all isolates showed 100% resistance to multiple β-lactams, and the class rates of aminoglycoside/tetracycline resistance genes aligned with the corresponding phenotypic resistance (62.0–65.0%). We further analyzed the effect of sex and age of giant pandas on the number of ARG subtypes detected on plasmids in each ESBL-producing *E. coli* strain. It was found that the number of ARG subtypes detected on plasmids in different sex and age showed no significant difference (*P* > *0.05*) (Fig. 5B, Fig. 5C).

**Fig. 6:**
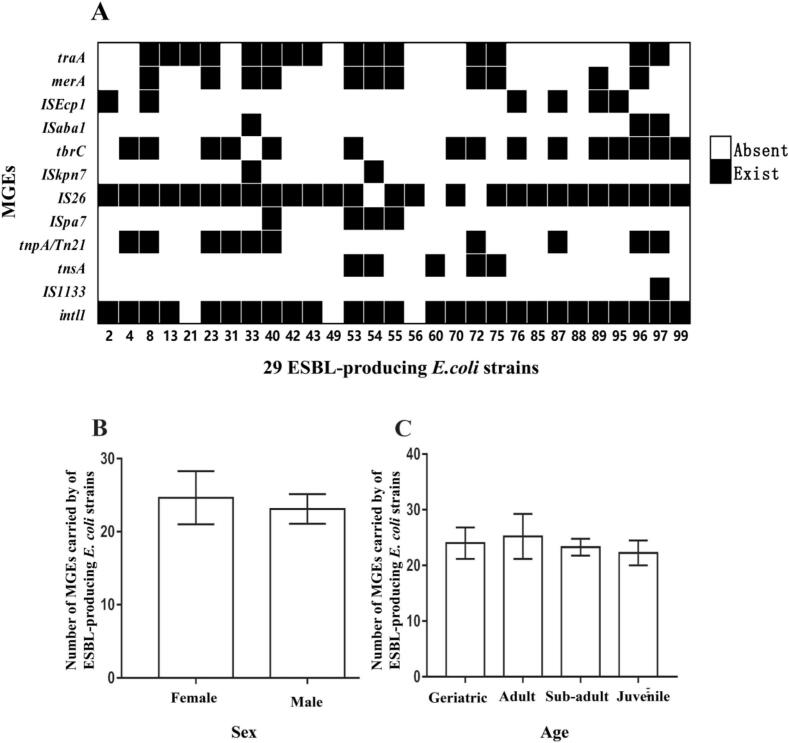
MGEs profiles of 29 ESBL-producing *E. coli* isolated from giant pandas. A. Distribution of MGEs in 29 ESBL-producing *E. coli* strains. The abscissa represents the individual isolates, and the ordinate represents 12 subtypes of MGEs. B. Effect of sex of giant pandas on the number of MGEs in each giant panda-derived ESBL-producing *E. coli*. C. Effect of age of giant pandas on the number of MGEs in each giant panda-derived ESBL-producing *E. coli.* Data were analyzed by one-way ANOVA using SPSS 22.0. No significant differences were observed between sexes or among age groups (*P* > 0.05).

The WGS results of MGEs showed that a total of 12 subtypes of MGEs were detected in 29 ESBL-positive strains (Fig. 6A), including *IS26* (89.7%, 26/29), *intI1* (89.7%, 26/29), *traA* (51.7%, 15/29), *tbrC* (51.7%, 15/29), *merA* (37.9%, 11/29), *tnpA/Tn21* (34.5%, 10/29), *ISEcp1* (20.7%, 6/29), *tnsA* (17.2%, 5/29), *ISpa7* (13.8%, 4/29), *ISaba1* (10.3%, 3/29), *ISkpn7* (6.9%, 2/29), *IS1133* (3.4%, 1/29) (Fig. 6A). We further analyzed the effect of sex and age of giant pandas on the number of MGE subtypes detected in each giant panda-derived ESBL-producing *E. coli*. It was found that there were no significant differences in the MGEs number based on the age and sex of the subject (*P* > 0.05) (Fig. 6B, Fig. 6C).

### 3.5 Molecular epidemiology analysis in the ESBL-producing *E. coli* based on the MLST

**Fig. 7:**
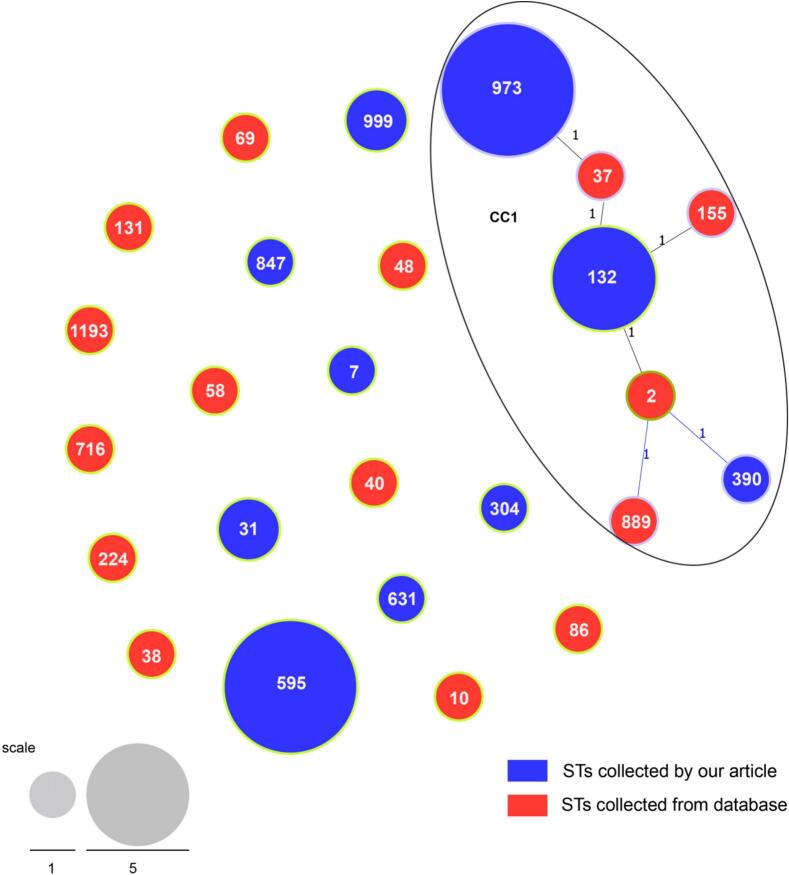
Molecular epidemiology analysis of the ESBL-producing *E. coli* based on the MLST through goeBURST distance algorithm. STs were symbolized by dots, the size of a dot corresponded to the number of isolates belonging to an ST. The scale is shown in the figure: a small dot represents 1 isolate, and a large dot represents 5 isolates. Dots of different colors indicate different sample sources, blue indicates *E. coli* STs collected in this study, red indicates the *E. coli* STs used for comparative analysis from the database, and the number marked on the line indicates the number of different alleles between the two subtypes. The colour around the ellipse indicates the status of each ST within the clonal complex (CC), light green indicates group founder, dark green indicates sub-group founder, light blue indicates common node. (For interpretation of the references to colour in this figure legend, the reader is referred to the web version of this article.)

We used MLST typing to understand the molecular epidemiology of the 29 ESBL-producing *E. coli* strains isolated from different giant pandas. A total of 10 sequence types (STs) were identified among the 29 ESBL-producing *E. coli* isolates by MLST, of which one isolate was not assigned an ST, as the eight housekeeping genes of this isolate could not be matched to any existing ST in the Pasteur Institute MLST database. ST595 and ST973 were the predominant sequence types (both *n* = 7), followed by ST132 (*n* = 5), ST999 (*n* = 2), ST31 (n = 2), ST7 (*n* = 1), ST304 (n = 1), ST390 (n = 1), ST631 (n = 1), ST847 (n = 1). Clonal structure analysis showed that the molecular epidemiology of the 29 ESBL-producing *E. coli* isolates was highly diverse; however, one clonal complex (CC1) was identified, namely CC1 (ST889, ST155, ST2, ST37, ST973, ST132, ST390), which was represented by an ellipse in the figure 7. In CC1, ST132 is the group founder, suggesting that this ST may be an ancestral ST from which other STs evolved, while ST2 is a subgroup founder (Fig. 7).

## 4 Discussion

Extended-spectrum β-lactamase seriously affects the therapeutic effect of β-lactam antibiotics, which has become a serious problem in human medicine and veterinary clinics [19], [20]. The prevalence rate of ESBL-producing *E. coli* strains isolated from giant pandas in this study was 29.0%, which was far lower than that in livestock and other captive wildlife animals. This prevalence rate of ESBL-producing *E. coli* strains from chickens in Qingdao area, rabbits in Sichuan Province, and tiger isolates in Siberian tiger gardens was 83.1%, 71.1% and 70.0%, respectively [21], [22], [23]. The lower prevalence of ESBL-producing *E. coli* in giant pandas may be explained by the less frequent use of antibiotics compared to humans and domestic animals, as well as their relatively uniform bamboo-based diet [24]. Compared with the previously reported prevalence of 8.0% in giant pandas during 2020–2021 [15], the prevalence of ESBL-producing *E. coli* in the present study has increased to 29.0%. This increase may be associated with multiple factors, including antimicrobial selection pressure, close contact among captive individuals, environmental exposure within the breeding facility, and the horizontal transmission of ARGs among intestinal bacteria. In addition, the long-term captive management environment may facilitate the persistence and dissemination of antimicrobial-resistant bacteria. These findings highlight the importance of continuous surveillance of ESBL-producing *E. coli* and the prudent use of β-lactam antibiotics in captive giant panda populations.

The spread of β-lactamases is frequently linked with plasmid-mediated ESBLs, specifically the *CTX-M* family [25]. The data obtained in this study indicated that *CTX-M* and *TEM* variants were the predominant genotypes, which was consistent with our previous reports of ESBL-producing *K. pneumoniae* strains isolated from giant pandas [26]. Zhou et al. [27] and Wan [28] also reported that ESBL-producing *E. coli* carrying *CTX-M* gene were detected in captive giant pandas. These findings suggest that *bla*_CTX-M_ may be the main prevalent ESBL-encoding genes in captive giant pandas. Furthermore, our results were similar to that described in previous studies, where *TEM* and *CTX-M* subtypes were the main families of ESBL [29], [30]. In addition, some genotypes of ESBL-producing *E. coli* isolated from giant panda were found in the study, such as *SHV, OXA,* but the *SHV* genosubtype was not detected in previous report of giant panda [27], and the *OXA-*genotype has been reported to be predominantly present in *Pseudomonas aeruginosa* and *Acinetobacter baumannii* [31], which indicates that the genotypes of ESBL-producing *E. coli* isolated from giant panda have become increasingly diverse, suggesting a potential increase in antibiotic resistance complexity in this species. The ESBL genotype is associated with the resistance phenotype, the *bla*_CTX-M_ enzyme is a plasmid-mediated class A β-lactamase, characterized by high hydrolytic activity against cefotaxime, so it is named cefotaximase, which is consistent with the susceptibility testing results obtained in this study. It is worth noting that a total of 19 different ESBL genotypes were detected in the study through WGS. Eighteen of these were located on the plasmids of bacterial strains. This substantially increases the potential for HGT of ESBL-producing *E. coli*.

In this study, 29 ESBL-producing *E. coli* strains isolated from the feces of giant panda from Chengdu showed different degrees of resistance to 34 antimicrobials, especially high resistance to AMX (100.0%), CXM (100.0%), CTX (100.0%) and AM (100.0%). When compared with a previous study that analyzed 84 *E. coli* strains from Chengdu in 2020 [32], our results showed increased resistant proportions of *E. coli* isolates to AMX (from 81.0% to 100.0%), AM (from 69.1% to 100.0%), CTR (from <50.0% to 93.1%), C (from <50.0% to 62.1%), FON (from <50.0% to 58.6%), TE (from <50.0% to 65.6%), SXT (from <50.0% to 55.2%), TMP (from <50.0% to 55.2%), and CIP (from <50.0% to 58.6%). However, the antimicrobial susceptibility test results showing high resistance to β-lactam antibiotics in this study are consistent with Liu et al.'s research of ESBL-producing *E. coli* strains isolated from the feces of giant panda [17], which indicates that the ESBL-producing strains have a wider resistance spectrum than non-ESBL-producing strains, especially β-lactam antibiotics. Fortunately, all 29 ESBL-producing *E. coli* strains in this study were sensitive to MEM, FOX and CTC, and these three antibiotics may be considered for the treatment of bacterial infections. In addition, we found that the number of antimicrobial resistances in juvenile giant pandas was significantly higher than that of adult and sub-adult giant pandas, which was consistent with the findings that most ARGs had a higher abundance in juveniles than in adult and geriatric in Mustafa et al.'s research [33]. This variation suggests that the association of gut bacteria with age and immunity varies with age, and may be either negative or positive [34], [35], [36]. Therefore, clinical medication also needs to consider the age factor of individual giant pandas.

The relationship between ARGs and phenotypic resistance is complex, as multiple regulatory mechanisms (e.g., post-transcriptional modifications, cellular metabolic state) can influence gene expression and final resistance levels [37], [38]. Nevertheless, in the present study, a high concordance between ARG carriage and phenotypic resistance was observed for most antibiotic classes, especially β-lactams, aminoglycosides, and tetracyclines (as summarized in Results). This consistency may be attributed to the fact that the majority of detected ARGs (including ESBL genes like *CTX-M-4*) were located on plasmids and were likely constitutively expressed. WGS enabled comprehensive and accurate detection of ARGs, which is essential for understanding resistance potential even when occasional discrepancies occur. This study comprehensively characterized the ARGs carried by 29 ESBL-producing *E. coli* strains through WGS, and the obtained results of ARGs detection were more comprehensive and accurate. The high prevalence of *IS26* (89.7%) and class 1 integrons (89.7%) in our ESBL-producing isolates indicates a strong potential for HGT. *IS26* is known to mobilize antibiotic resistance genes, particularly those encoding β-lactamases. The *intI1* gene captures and expresses gene cassettes, facilitating MDR spread. The presence of *traA* (51.7%) further supports plasmid-mediated dissemination of ARGs among giant panda gut bacteria.

WGS is a powerful approach for characterizing bacteria at the genomic level. Analyzing the structure and function of bacteria at the genetic level is an essential prerequisite for studying their physiological characteristics [39]. In this study, 120 different subtypes of ARGs were detected in 29 ESBL-producing *E. coli* isolates through NGS of whole genome sequencing, and at least 30 subtypes of ARGs were carried by every strain. One hundred twenty subtypes of ARGs detected in the study mainly belonged to β-lactamases, aminoglycoside and multi-drug resistance, which was consistent with the results of our antibiotic resistance phenotype test and also the previous study [40], [41]. In general, the antibiotic resistance phenotype is determined by the carrying and expression of ARGs [42]. ARGs carried by *E. coli* strains are closely related to the antibiotic resistance phenotype [43]. The 37 subtypes of ARGs such as *acrA*, *emrA* and *mdfA*, located on the chromosome of 29 ESBL-producing *E. coli* isolates, all had a high detection rate of 100%, which can lead to antibiotics inherent resistance and multiple resistance of giant pandas in clinical treatment; in addition, the detection results of tetracycline resistance genes showed that *tet(A)* and *tet(M)* were detected, while Zou et al. [44] detected *tet(A)* and *tet(B)* in *E. coli* isolated from giant pandas, and Wang et al. [45] detected *tet(B)* and *tet(D)* in *E. coli* from diarrhea of calves in Shanxi. In comparison, *tet(A)* may be the main genotypes of tetracyclines in *E. coli* strains isolated from giant pandas.

Antibiotic resistance can be obtained through horizontal transfer of ARGs via MGEs including plasmids, transposons, insertion sequences, integrons, etc., which also play an important role in the dissemination of ARGs among *E. coli* strains [27]. A total of 12 types of MGEs were detected in the 29 *E. coli* strains in this study, among which *IS26* (89.7%, 26/29) and *intI1* (89.7%, 26/29) accounted for a relatively high proportion, followed by *traA* (51.7%, 15/29) and *tbrC* (51.7%, 15/29). Insertion sequences (ISs) are the smallest transposable elements, which can be transferred between integrons, transposons, plasmids, and chromosomes, thereby contributing to the spread of drug resistance and activating downstream gene expression. *ISPa7* has been reported in *Pseudomonas aeruginosa*, ISAba1 in *Acinetobacter baumannii* and *IS26*, *IS903*, *ISEcp1*, and *ISCR* in *Escherichia coli* [46], [47], [48], [49]. In this study, *ISPa7*, *ISAba1*, *IS26* and *ISEcp1* were detected in ESBLs-producing *E. coli*, which indicates that some insertion sequences have been transferred and disseminated among bacterial species. The *traA* gene and *tbrC* gene are the two main genetic markers of the conjugative plasmid, which encode bacterial pili, essential components for bacterial conjugation. It is worth noting that the detection rates of *IS26* and *traA* in our study were higher than those in the study by Zhu et al. [32] on MGEs in *E. coli* strains isolated from giant pandas at the CRBGP in 2020 (*P* < 0.01). Integrons are genetic units for mobile gene capture and expression, and can also mobilize and capture ARGs of *E. coli* encoded on MGEs by gene cassettes, which is one of the important reasons for the emergence of MDR strains. The *intI2* and *intI3* genes were not detected in our study. Many studies have shown that class 1 integrons are more common in Gram-negative bacteria than class 2 or class 3 integrons [50], [51]. In our study, the proportion of isolates containing the *intI1* gene (89.7%, 26/29) was higher than that previously reported in giant pandas [30].

MLST is a bacterial-typing method based on the sequences of internal fragments of seven or eight housekeeping genes. The MLST approach is commonly used to investigate the phylogeny and population structure of different bacterial isolates, which is helpful for investigating the molecular epidemiology and evolutionary studies of bacteria. In our study, 29 ESBL-producing *E. coli* isolates were divided into 10 STs, among which ST595 and ST973 (both with 7 strains) had the highest number of isolates, accounting for 24.1% of the total isolates; and ST132 (5 strains) had the second highest number of isolates, accounting for 17.24% of the total isolates; the remaining 7 STs contained 1–2 strains each, totaling 9 strains, accounting for 31.0% of the total strains. The above results indicated that ESBL-producing *E. coli* isolates had high genetic diversity.

Clonal structure analysis through goeBURST distance algorithm showed that there was one clonal complex, in which ST132 is the group founder. It indicated that although the molecular epidemiology of 29 ESBL-producing *E. coli* strains isolated from different giant pandas was highly diverse, some strains still had the same origin. At the same time, in order to understand the evolutionary relationship between *E. coli* STs in this study and other major STs of *E. coli* commonly found worldwide or in giant pandas, we added 9 important STs to our molecular epidemiology analysis (Fig. 7). The ST86, ST716, ST155 and ST40 were sequence types of pathogenic *E. coli* isolated from loose fecal samples of captive giant pandas with diarrhea [18]; ST973, ST132, ST595 and ST37 were the main sequence types of *E. coli* isolated from feces of healthy captive giant pandas in Li et al.'s research [52]. The ST973, ST595 and ST132 also were the main sequence types in our study, which belonged to the same CC1 as ST155 and ST37 in the above study. It indicates that some common STs of *E. coli* from giant pandas may be related. ST2 was the most prevalent ST in CTX-M-type ESBLs producing *E. coli* isolated from clinical settings [53]; ST889 with a high prevalence of CTX-M was found among ESBL-producing *E. coli* isolates from waterfowl in Hainan, China [54]. ST2 and ST889 in the above research had the same group founder ST132 with ST390 and ST973 in ESBL-producing *E. coli.* In our study, it indicates that there may be a relationship between sequence types and the *bla*_CTX-M_ genotypes of *E. coli* strains. *E. coli* ST131 is a worldwide pandemic clone, causing predominantly community-onset antimicrobial-resistant infection [55]; ST10 is a globally disseminated pathogen of both humans and wildlife and linked with many resistance mechanisms [56]. In contrast, the STs identified in the present study showed minimal overlap with these previously reported high-risk pathogenic and MDR lineages (Fig. 6).

## 5 Conclusion

In this study, the prevalence of ESBL-producing *E. coli* in captive giant pandas at the CRBGP was 29.0% (29/100), which is markedly higher than the 8.0% reported in 2020–2021. All 29 isolates exhibited 100% phenotypic resistance to AMX, AM, CXM, CTX, and CZ. A total of 120 ARG subtypes were detected. Nineteen different ESBL genosubtypes were identified, among which *bla*_CTX-M-4_ was the most prevalent (100.0%), followed by *bla*_SHV-1_ (96.6%), *bla*_CTX-M-1_ and *bla*_CTX-M-3_ (both 93.1%). High carriage rates of mobile genetic elements were observed: *IS26* (89.7%), class 1 integrons *intI1* (89.7%), and the conjugation-associated gene *traA* (51.7%). MLST analysis revealed 10 sequence types (STs) and one clonal complex (CC1) with ST132 as the group founder. ST595 and ST973 were the most common STs (each *n* = 7). Some STs (ST973, ST595, ST132) were shared with *E. coli* isolates from other giant pandas and other species, suggesting possible cross-species transmission. Additionally, juvenile giant pandas carried a significantly higher average number of antimicrobial resistances (mean number of resistance phenotypes per isolate) (24) than adults (15.9) and sub-adults (16.0). These findings collectively demonstrate an alarming rise in ESBL-producing *E. coli* with high-level MDR and a strong potential for HGT. Enhanced antimicrobial stewardship, routine surveillance, and infection control measures are urgently needed to limit the spread of these resistant bacteria in the captive giant panda population.

## CRediT authorship contribution statement

**Xia Yan:** Writing – review & editing, Writing – original draft, Methodology, Investigation. **Lucai Wang:** Writing – review & editing, Writing – original draft, Validation, Methodology, Investigation. **Huanrong Zhang:** Methodology, Investigation. **Mei Yang:** Methodology. **Bingyu Xue:** Validation. **Ruoshui Zhao:** Investigation. **Lin Li:** Methodology. **Junjin Xie:** Investigation. **Songrui Liu:** Methodology, Investigation. **Xueyang Fan:** Software. **Xiaoyan Su:** Writing – review & editing, Resources, Methodology.

## Ethical consideration

Fecal sample collection from captive giant pandas in the study was non-invasive and approved by the Institutional Animal Care and Use Committee (IACUC) of the Chengdu Research Base of Giant Panda Breeding (No. 2018017). The study was conducted in accordance with the Guide for the Care and Use of Laboratory Animals of the Ministry of Science and Technology of the People's Republic of China and the ARRIVE guidelines for reporting in vivo experiments where applicable.

## Funding

This research was supported by 10.13039/501100004829Sichuan Provincial Department of Science and Technology, China (project nos. 2024ZYD0132), Chengdu Park City Construction Authority, China (project nos. 202503KY0006 & 202503KY0008).

## Declaration of competing interest

The authors declare that they have no known competing financial interests or personal relationships that could have appeared to influence the work reported in this paper.

## Data Availability

Data will be made available on request.
